# Effect of Regular Yogic Training on Growth Hormone and Dehydroepiandrosterone Sulfate as an Endocrine Marker of Aging

**DOI:** 10.1155/2014/240581

**Published:** 2014-05-08

**Authors:** Sridip Chatterjee, Samiran Mondal

**Affiliations:** ^1^Department of Physical Education, Jadavpur University, Kolkata,West Bengal 700032, India; ^2^Department of Physical Education, Vinaya Bhavana, Visva Bharati, Santiniketan,West Bengal 731235, India

## Abstract

Growth hormone (GH) and dehydroepiandrosterone sulfate (DHEAS) secretion decline with advancing age and are associated with the symptoms of aging. Yogic texts claimed that regular practice of yoga may restore and maintain general endocrinological properties in the human body. *Objective of the Study*. To observe the effect of yogic training for twelve weeks on basal level of GH and DHEAS in middle aged group. *Method*. Forty-five untrained volunteers were divided into two groups, that is, yoga practicing (experimental: male 15, age 42.80 ± 7.43 yrs; female 8, age 44.75 ± 8.40 yrs) and waitlisted control group (male 15, age 41.67 ± 7.87 yrs; female 7, age 45.43 ± 7.00 yrs). The experimental group underwent combined yogic practices daily in the morning for 6 days/week for 12 weeks, whereas control group continued their usual routine activities. Standing height, body weight, body mass index, and basal level of GH and DHEAS were measured before commencement and after six and twelve weeks of yogic training period. The repeated measure ANOVA was used for data analysis. *Results*. 12 weeks of yogic training produces a significant increase in GH and DHEAS for both male and female groups as compared to their baseline data, whereas no as such changes were observed in the control group. *Conclusion.* Combined approach of graded yogic training may be beneficial for maintaining the basal level of GH and DHEAS in the human body, thus promoting healthy aging.

## 1. Introduction

Human growth hormone (GH) secreted from anterior pituitary has important roles in growth of almost all tissues, metabolism, and changes in body composition [1–3]. Dehydroepiandrosterone sulfate (DHEAS), secreted by the adrenal cortex, acts in the human body as a neurosteroid, cardioprotective, antidiabetic, antiobesity, and immunoenhancing agent. It is also reported as youth hormone [4–6].

Basal level of GH and DHEAS declines with advancing age and is reported as antiaging hormones [7–11]. Nonpharmachological replacement of these hormones by natural stimulus like yoga may be a strategy for delaying the onset of aging [11]. GH and DHEAS showed a slower decline in active individual than the inactive peers [4]. Regular exercise habit induces the secretion pattern of GH and DHEAS throughout the lifespan [12–16].

It is reported in the ancient yogic texts that regular practice of yoga may delay aging process [17–23]. All traditional schools of yoga advocate a movement towards perfection of the body, perfection of the mind, or that of both. Today yoga is popular not so much as a system of philosophy but as a system of practical discipline to improve physical, physiological, psychological, and spiritual health [24–29]. Researches on GH, DHEAS, and yogic training in the perspective of aging are very few.

Jevning et al. [30] studied plasma prolactin and growth hormone before, during, and after transcendental meditation (TM) and found that GH concentration was unchanged in both TM and rest group. In another study, changes in baseline levels and responses to laboratory stressors were examined for growth hormone before and after 4 months of either the TM technique or a stress education control condition. Growth hormone increased after 4 months of TM meditation in response to laboratory stressors [31]. Plasma levels of growth hormone concentrations fell slightly (insignificant) in experienced meditators and nonmeditators groups during and after meditation/relaxation period, the trend being more apparent in meditators [32]. The long-term practice of TM and TM Sidhi Programme for consecutive weeks (initial and after 5, 49, 115, and 167 weeks) showed a progressive insignificant decrease of growth hormone [33]. Schell et al. [34] reported that there was no substantial growth hormone difference between yoga practice group and control group. Dehydroepiandrosterone sulfate (DHEAS) was measured in 270 male and 153 women who were experienced practitioners of the TM and TM Sidhi Programme. The mean DHEAS levels were found higher in all the age groups compared with age-sex matched nonmeditators [35]. Long-term combined practice of yoga (asana, pranayama, and meditation) produces an insignificant increase of DHEAS level compared to control group [36].

Most of the above mentioned researches were mainly conducted to observe the effect of yoga on single intervention (meditation) except the study of  Vera et al. [36]. The principle of “Holistic approach of Yogic Science,” that is, the body as an integrated (whole) matter rather than a series of systems, has been considered in the present study. Yogic practice will also be useful if the intensity and duration of the training are maintained properly.

Therefore, in this study, the researcher attempted to observe the combined effect of regular graded yogic training for twelve weeks on basal level of GH and DHEAS in middle aged group.

## 2. Materials and Methods

### 2.1. Study Location

The present study was carried out in the Department of Physical Education, Visva-Bharati University, Santiniketan, Birbhum, West Bengal, India.

### 2.2. Subjects

**Figure 1:**
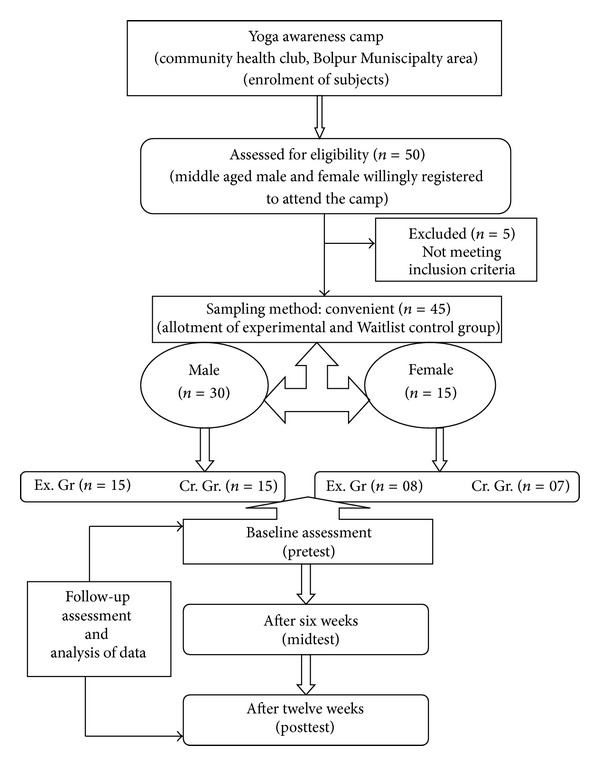
Trial profile.

A “Yoga Awareness Camp” was organized jointly by the researcher and one of the renowned community health clubs in Bolpur (Santiniketan) Municipality area for collection of data. Fifty middle aged male and female willingly registered their names to attend this yoga camp. Forty-five healthy middle aged persons were able to meet the screening criteria. Subjects' age range was between 35 and 55 years, belonging to almost the same socioeconomical background. They were recreationally active but not specifically acquainted with the yogic practices before. Five subjects were excluded from the study due to major injury or illness. Subjects were free to withdraw themselves from the training at any point during the study (Figure 1).

### 2.3. Study Design

In the present study the convenient sampling method was adopted because there were some couples who were willing to volunteer themselves in this study if they got the opportunity to practice together. Considering their requests, the subjects were divided on the first come first serve basis. So, according to the serial of the registration, 23 (male 15, age 42.80 ± 7.42 yrs; female 8, age 44.75 ± 8.40 yrs) middle aged healthy volunteers served as experimental group and 22 (male 15, age 41.67 ± 7.87 yrs; female 7, age 45.43 ± 7.00 yrs) age-sex matched subjects were in the waitlisted control group. The experimental group underwent yogic practices, whereas waitlisted control group maintained usual routine activities. To meet the purpose of the study, both the experimental and waitlisted control group subjects were assessed at baseline (pretest), after six weeks (midtest) and twelve weeks (posttest) accordingly. The “Board of Studies,” Department of Physical Education, Visva-Bharati University, went through the whole procedure of this study and forwarded this to the institutional (university) research board. Finally, the university research board approved the study. The aims and objectives of this study were thoroughly discussed with all the subjects, and then a standard informed consent forms were signed by the subjects.

### 2.4. Assessments

Chronological age, standing height, body Weight, body mass index (BMI), basal level of plasma growth hormone (GH), and dehydroepiandrosterone sulfate (DHEAS) were measured for this study. All the data were collected according to the guidelines of standard scientific manual. Instruments were calibrated every day before the experiment and standard procedural steps were followed. The extreme scores (highest and lowest), if any, were discarded.

### 2.5. Hormonal Assay

Basal level of GH and DHEAS was measured by enzyme-linked immunosorbent assay method (ELISA).

*Specimen Collection and Preparation*. Following an overnight (12 hrs) fasting, venues blood samples were taken via a disposable plastic syringe inserted into an antecubital forearm vein. Blood (5 mL) was allotted to colt for one hour in a plain test-tube (Burocell) and then centrifuged (REMI) at 30°C for 5 minutes at 6000 Rev min^−1^ to separate the serum from the cells. Before proceeding with the ELISA test, (Monobind Inc., Lake Forest, CA 92630, USA) reagent (GH, Product Code: 1725-300; DHEAS, Product Code: 5125-300) and specimen serum were brought to room temperature (20–27°C).

*GH Assay*. Reagent preparation*:* wash Buffer: 0.300 mL wash buffer was mixed with 1000 mL distilled water and kept at room temperature (20–27°C). Working substrate solution*:* the contents of the amber vial labeled solution “A” poured into the clear vial labeled solution “B,” mixed and labeled accordingly and stored at 2–8°C.* Test *Procedure*:* formatting the microplates' wells for each serum reference. 0.05 mL specimen serum was taken by the pipette (0–50 mL) and given into the assigned wells. 0.100 mL enzyme reagent solution was added to all wells and swirl the microplate gently for 20–30 seconds to mix and then incubated for 60 minutes at room temperature. Manually wash the microwells by the prepared wash buffer for three times and dry them with tissue paper. After that 0.100 mL substrate solution was add to all wells and incubated (20–27°C) for 15 minutes. Reagents were added in the same order to minimize reaction time differences between wells. Immediately after that, add 0.050 mL of stop solution to each well and gently mix it for 15–20 seconds. Recording of results*:* the microplates were passed under the ELISA reader machine and results were recorded in ng/mL.(Manual, Monobind Inc. Lake Forest, CA 92630, USA). 

*DHEAS Assay*. Reagent preparation*:* wash Buffer: 0.300 mL wash buffer was mixed with 1000 mL distilled water and kept at room temperature (20–27°C). Working substrate solution*:* the contents of the amber vial labeled solution “A” poured into the clear vial labeled solution “B,” mixed and labeled accordingly and stored at 2–8°C. Test procedure*:* formatting the microplates' wells for each serum reference. 0.010 mL specimen serum was taken by the pipette (0–50 mL) and given into the assigned wells. 0.100 mL enzyme reagent solution was added to all wells and swirl the microplate gently for 20–30 seconds to mix. Anti-DHEAS biotin reagent should be added to all wells and swirl the microplate again for 20–30 seconds to mix and finally incubate for 30 minutes at room temperature. Manually wash the microwells by the prepared wash buffer for three times and dry them with tissue paper. After that 0.100 mL substrate solution was add to all wells and incubated (20–27°C) for 15 minutes. Reagents were added in the same order to minimize reaction time differences between wells. Immediately after that, add 0.050 mL of stop solution to each well and gently mix it for 15–20 seconds. Recording of results*:* the microplates were passed under the ELISA reader machine and results were recorded in ng/mL. (Manual, Monobind Inc. Lake Forest, CA 92630, USA).

### 2.6. Training Protocol

The yoga training protocol was formulated according to the health and physical ability of the subjects. The yoga intervention consisted of Suryanamaskara (sun salutation) or Shithilikarana (loosening) practices, Kriyas (cleansing process), Asanas (static and dynamic postures), Pranayamas (breathing practices), and Dhyana (meditation). Detailed yogic practices are scheduled according to the demand of the yogic science and to fulfill the objectives of the study. Progressive training load method [37] was applied in the form of duration, repetitions, and volume from 1st week to the end of the training (twelve weeks) period (Tables 1, 2, 3, 4, 5, and 6). From 8th week onward three types of practice combination were used. Each of these combinations was practiced two days in a week. There are few yogic techniques which cannot be practiced regularly, whereas another technique needs longer time to practice. So to justify the training load and maintain it accordingly the present researcher adopted this combination schedule. Waitlisted control group was given no specific intervention and continued with their routine activities. They attended “health and positive mental attitude awareness” class in a day per week. A general record book was also maintained to note their daily activity level and lifestyle. Details of the combination were as follows.

Combination 1: Kriya (cleansing process) 30 mins; Suryanamaskara (sun salutation) 10 mins; Asana (posture) 35 mins; Pranayama (breathing practices) 20 mins; and dhyana (meditation) 10 mins. 

Combination 2: Suryanamaskara 10 mins; Kriya 10 mins; Asana 35 mins; Pranayama 25 mins; and dhyana for 25 mins. 

Combination 3: Suryanamaskara 15 mins; Kriya 15 mins; Asana 45 mins; Pranayama 25 mins; and dhyana for 5 mins (Tables 7, 8, and 9).

### 2.7. Statistical Procedure

In the present study the repeated measures analysis of variance (RM ANOVA) was used for data analysis [38]. Repeated measures ANOVA are an extension of paired *t*-tests. Here, RM ANOVA was used to test for (i) significant differences between the assessments, that is, at baseline (pretest), after six weeks (midtest), and after twelve weeks (posttest), and this was a within subjects factor denoted by time and (ii) differences between the groups (experimental (yoga) and waitlist control) and this was a between subjects factor, and the test for a time by group interaction provides a global test for an intervention effect. Postdata were compared to pre- and middata of respective group using post hoc analysis with Bonferroni adjustment. The level of significance was set at 0.05 levels (*P* < 0.5). Simple percentages (%) were also calculated from the mean value to see the quantitative changes of the yogic training. Repeated measures “Analysis of Variances” (RM ANOVA) are one in which multiple measurements on the same experimental subjects comprise the replicate data [39].

## 3. Results

### 3.1. Baseline Characteristics

Before commencement of the yogic training baseline difference between experimental and control group for the selected variables was calculated (independent *t*-test) and insignificant difference was observed for both male and female group (Table 10).

*General Health Variables*. The body weight and BMI of experimental group (male and female) fall significantly (*P* < 0.001) after twelve weeks of graded yogic training compare to baseline where as in the control group no such changes were observed during these twelve weeks (Table 2).

### 3.2. Endocrinological Variables

**Figure 2:**
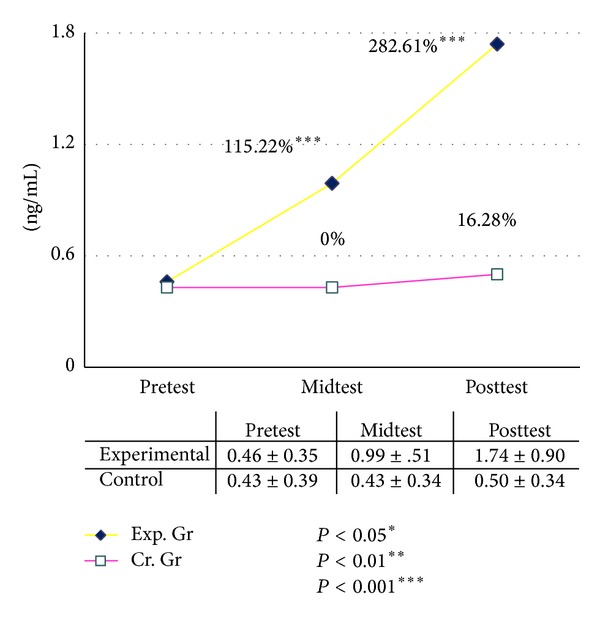
Human growth hormone (hGH) male.

**Figure 3:**
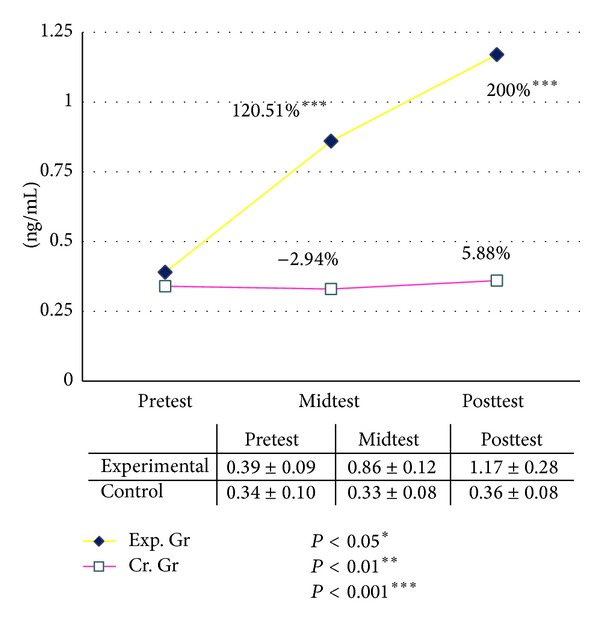
Human growth hormone (hGH) female.

In the present study the basal level of serum GH was increased significantly after six and twelve weeks of graded yogic training compared to baseline, whereas no such changes were observed in the waitlist control group for both male and female (Table 11). The improvements were recorded 115% after six (*P* < 0.001, paired *t*-test, pretest versus midtest) and 282% after twelve weeks (*P* < 0.001, paired *t*-test, pretest versus posttest) in male group as compared with the baseline value. In the female group the GH increased 120% after six (*P* < 0.001, paired *t*-test, pretest versus midtest) and 200% after twelve weeks (*P* < 0.001, paired *t*-test, pretest versus posttest) of yogic training as compared with the baseline value, whereas changes were negligible in control group during this twelve weeks (Figures 2 and 3). The ANOVA results are summarized in Table 12.

**Figure 4:**
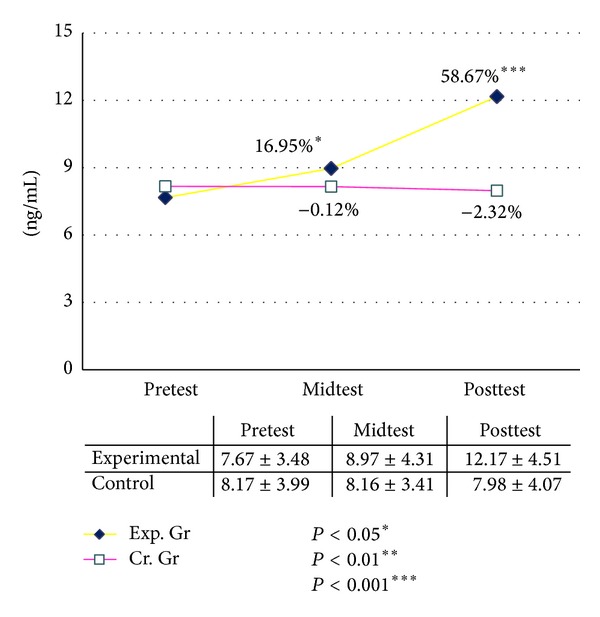
Dehydroepiandrosterone sulfate (DHEAS) male.

**Figure 5:**
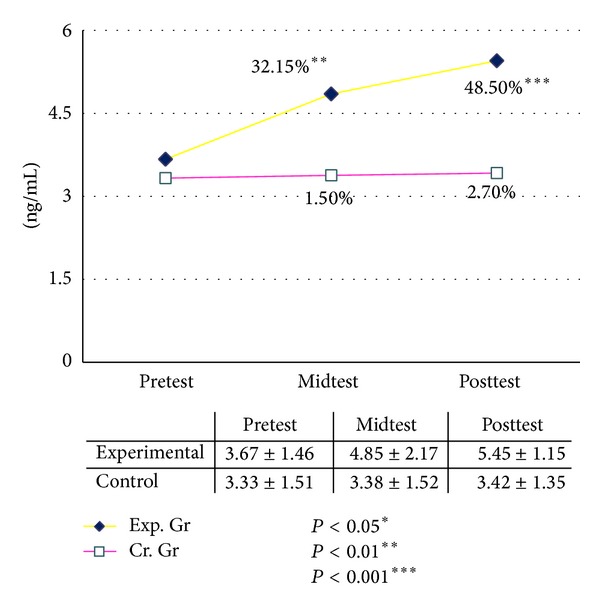
Dehydroepiandrosterone sulfate (DHEAS) female.

The basal level of serum DHEAS was increased significantly after six and twelve weeks of graded yogic training compared to baseline, whereas no such changes were observed in the waitlist control group for both males and females (Table 11). The improvements were recorded as 16.95% after six (*P* < 0.04, paired *t*-test, pretest versus midtest) and 58.67% (*P* < 0.001, paired *t*-test, pretest versus posttest) after twelve weeks in male group as compared with the baseline value. In the female group it was increased by 32.15% (*P* < 0.01, paired *t*-test, pretest versus midtest) after six and 48.50% (*P* < 0.001, paired *t*-test, pretest versus posttest) after twelve weeks of yogic training as compared with the baseline value, whereas changes were negligible in control group during this twelve weeks (Figures 4 and 5). The ANOVA results are summarized in Table 13.

## 4. Discussion

The main findingof the present experiment is that the combined approach of graded yogic training is positive and associated with increased basal level of GH and DHEAS in the blood. It also appears from the results that combined yogic practices are a productive means for the maintenance of body weight in the middle age. BMI is a standard (WHO) index for the expression of general health. This numerical value may indicate malnutrition in the lower range and obesity in the higher range. The change of BMI in the experimental group was for the decrease of body weight which supporta the result of previous studies [40–44].

All endocrine glands are subject to the effects of aging [16]. GH output is relatively low before puberty but with sexual maturations and adolescence there is a period of high GH output and accelerating somatic growth [45]. GH secretion and serum GH concentrations fall, both basally and in response to stimuli. In older subjects the decrease in growth hormone secretion is known to cause a reduction of protein synthesis, a decrease in lean body mass and bone mass, and a decline in immune function [46]. It can be revealed that previous research studies were conducted to observe the effect of yoga on GH response. Growth hormone concentration was unchanged before, during, and after transcendental meditation (TM) in the long-term transcendental meditation practitioner group, restudied controls and control group [30]. GH concentrations fell slightly in the TM group and in the control group during the postmeditation/relaxation period, the trend being more apparent in meditators [32]. Werner et al. [33] reported that a progressive decrease in GH level occurred over three years of transcendental meditation programme. MacLean et al. [31] observed the baseline and responses to laboratory stressors for growth hormone either after the 4 months of TM programme or a stress education control programme. Growth hormone increased after 4 months of TM meditation in response to laboratory stress session.

Most of the above reports of yoga on growth hormone have dealt with transcendental meditation. Transcendental meditation gives rise to a unique state of deep rest by marked reductions in resting heart rate, respiratory rate, oxygen consumption, and metabolic activity [47–49]; increased cerebral blood flow [30, 49, 50] may be responsible for the decrease of growth hormone in the human body. These results of TM technique also suggest meditation may produce mental alertness with physiological relaxations.

In contrast to our pilot study, it was observed that six weeks combined yoga intervention produce an insignificant increase in the basal level of GH [51]. Also in this present study, maintaining all the methodological formalities except randomized control trial, again it was observed that GH level increased significantly after six and twelve weeks of yogic practices for male and female middle aged group. In the graded yogic training schedule there were suryanamaskara (sun salutation), shithilikarana (loosening) practices, and asanas (physical postures) which were reported as moderate aerobic type of exercises [52–56] and may positively influence the basal level of GH in the plasma blood. There are many exercise and sports scientists who have reported the same that regular moderate exercise habit may improve/increase basal levels of GH in the human subjects [13, 15, 57] which positively support our findings.

On the other hand different cross-sectional studies also provided some important mechanisms. GH and Insulin like growth factor-I (IGF-I) influence the function of the hippocampus, a brain structure important for the maintenance of cognitive functions such as learning, perception, and memory. Several studies mention that GH or IGF-I deficiency is associated with the deterioration of cognitive functions [58–60]. Cognitive abilities have been improved after regular practice of yoga [61–67]; this central adaptation mechanism may influence the improvement of GH level. VO_2_ as a marker of fitness is an independent predictor of peak GH secretion. Hatha yoga practices increased the physical fitness [68–71] and aerobic capacity after immediate practice of asana [56, 72, 73] that may influence the increase of GH levels in the blood. Insulin like growth factor-I (IGF-I) and GH production are positively correlated; increase of IGF-I after moderate exercise may be another possible mechanism for the improvement of GH secretion [74]. Deep sleep increase GH secretion [75], meditation, is an alert full deep resting state associated with high alfa waves, very similar to the stage of deep sleep that may increase GH secretion. There was an established relationship between decrease cortisol level and increased GH level [14, 76]. Cortisol level has been decreased significantly after practice of yoga may be another possible cause of increasing GH. The levels of GH have positively correlated with the secretion of DHEA/DHEAS [4]; in the present study the DHEAS secretion increased significantly after yogic training may have a positive impact on increased basal level of GH.

Dehydroepiandrosterone sulfate (DHEAS) is secreted from the deep layer of adrenal cortex and is called zona reticularis [77]. The blood level of DHEAS peaks at approximately 20–25 years of age and declines rapidly and markedly after the age of 30 years [78].

Dehydroepiandrosterone (DHEA) increased in six and decreased in two healthy subjects after immediate practice of cobra posture (bhujangasana) in a group of 22–25 yrs aged healthy subjects [79]. Glaser et al. [35] measured DHEAS level in transcendental meditation practitioner of different age group. They compared all the groups with age matched men and women and found 6% elevation in the 42–44-year-age group; 13% in the 45–49-year group; 54% in the 50–55-year group, whereas in the female group elevation was reported as 28% in the 35–39-year group; 28% in the 40–44-year group; 34% in the 45–49-year group; 54% in the 50–54-year group; and 29% in the 55–59-year group. Vera et al. [36] reported that DHEAS level in the yoga group was 137.15 ± 53.08 ug/dL and, in the control group, it was 118.18 ± 58.86 ug/dL. The reports of the above studies were a very close proximity with this present research. The combined (Kriya, Suryanamaskara, Asana, Pranayama, and Meditation) practice of yoga produce a significant improvement of serum DHEAS level after six and twelve weeks of graded yogic training.

Convenient sampling (not randomized control trial) and the fact that no residential camp was conducted can be considered to a certain extent as limitations of the study. Within the limitations of the study, the researcher would like to present the prospective mechanisms of yoga for increasing the following basal level of GH and DHEAS after regular graded combined yogic practices for a period of twelve weeks.

- Mind and body are not separate entity in yoga. The practice of Kriya, Suryanamaskara and Asana (physical level), Pranayama (psychophysiological level), and Meditation (psychoneurological level) integrate and harmonise mind and body to provide an ideal neuroglandular adjustment within the individual and may positively stimulate the GH and DHEAS secretion pattern in the middle aged persons.
- Kriyas or cleaning processes (Kapalbhati, Agnisara, Uddiyan Jalaneti, Sutraneti, and Vamandhauti) bring in control over the autonomic nervous system function [17]. This may send positive feedback to the hypothalamus-pituitary axis which could influence the basal GH production in the blood. Kapalbhati, Agnisara, and Uddiyan give vigorous abdominal movements and an automatic abdominal massage may preserve the health of adrenal gland and thus improve DHEAS concentration in the blood. Kriyas bring about a widened range of adaptability of the tissues forming the various systems and organs as also raise the threshold of their reactivity. Voluntary control is established on different reflexes through the cleaning processes. Autonomic and proprioceptive neuromuscular reactions seem to have an important bearing in bringing about these results [80, 81].
- Suryanamaskara or salutation to the sun is an important yogic practice which has been handed down from the sages of Vedic time. Suryanamaskara is almost a complete sadhana in itself [82]. It is a series of dynamic movements (12 counts) of forward and backward spinal bends and stretches with body and breathing awareness improve major muscle group in the body, strengthen joint structure and range of motion, digestion, circulation, aerobic capacity, body's circadian rhythms, nourishment, and stimulation of the nerves [83–87]. With this it is also equally activate and stimulates all the glands in the body including pituitary and adrenal to get a positive neuroendocrine feedback for maintaining a healthy secretion of GH and DHEAS.
- Cultural asanas (static physical posture with internal body awareness) provides a constant supply of proper nutrition to the tissues and the internal secretion of the endocrine glands [88]. The hypothalamopituitary axis is best taken care of by antigravitational postures, for example, Sarvangasana, Viparitkarana Asana, and Sirshasana. So far as the adrenals are concerned, Pavanmuktasana, Halasana, Paschimottanasana, Bhujanga, Salvasana, Dhanurasana, Usthrasana, Bakrasana, and Yogmudra Asana are capable of preserving their health which may positively influence the basal plasma level of DHEAS in the body. Erect position of the spine in meditative asanas, for example, Padmasana and Vajrasana, eliminate the possibility of the compression of the abdominal viscera and also free the mind from the burden of the body, richer blood supply to the pelvic region, and minimum production of carbon dioxide in the body due to laser muscular involvement produce a parasympathetic dominance which may indirectly influence the GH and DHEAS basal level in the body.
- In the machinery of human body a liberal supply of oxygen to the circulating blood is of supreme importance for the health of an individual. This supply is effectively improved by means of pranayama (scientific breathing practices). This richer and more liberal blood supply brought to the endocrine glands makes them healthier [89]. Regular practice of different forms of Pranyama (Anulome-vilome, Surya Vedhana, Chandra Vedhana, Ujjai, Bhastrika, Shitali, and Sitkari) increases psychophysiological relaxation by quieting and calming the mind, decreases sympathetic tone, and enhances parasympathetic activation [90–92] which may positively influence the basal level of GH and DHEAS concentration in the blood.
- Meditation is believed to gradually diminish sympathetic dominance, resulting in a better balance between the sympathetic and the parasympathetic activity [93]. It also brings about a hypometabolic state [94]. By modifying the state of tension and anxiety, mediation reduces stress induced sympathetic over reactivity [93, 95, 96]. Thus a decrease in sympathetic response and ability to overcome stress can be a possible reason for the improvement in GH and DHEAS in the plasma blood.

## 5. Conclusion

Finally from the findings of the present study and from the above elaborate discussion, it can be concluded that combined approach of yoga (Kriya, Suryanamaskara, Asana, Pranayama, and Meditation) significantly increases the basal level of GH and DHEAS in the blood, thus contributing in promoting healthy aging. The results of the present study also support the claim made by the seers and sages of India in the ancient yogic texts.

## Figures and Tables

**Table 1:** Detailed yogic practice protocol for 1st week (45 minute-schedule).

Name of yogic practices	Execution time/intensity	Repetitions/frequency	Recovery time/density	Total time/volume
Suryanamaskar (12 counts): (1) Pranamasana, (2) Hasta Uttanasana, (3) Padahastasana, (4) Aswa Sanchalanasana, (5) Parvatasana, (6) Ashtanga Namaskar, (7) Bhujangasana, (8) Kurmasana, (9) Tulasana, (10) Aswa Sanchalanasana, (11) Padahastasana, and (12) Hasta Uttanasana (3 days/week) OR	1.5 × 5 = 7.5 min	5 times	30 sec × 5 = 2.5 min	10 min
Shitilikarana Practices, and Tadasana Practices (3 days/week)	4 × 2 = 8 min	2 times	1 × 2 = 2 min	

Kriya: Kapalbhati	30 strokes @ 1 stroke/sec = 2.5 min	5 times	30 sec rest in between each round = 2.5 min	5 min

Asana: Ardhahalasana, Ekpadapabanmuktasana, Bhujangasana, Ardhasalvasana, Yogmudra, Bakrasana, Janusirshasana, Ardhakati Chakrasana, Trikonasana, and Vadrasana	Holding time: 5 sec 10 Asana × 1.5 min = 15 min	1 times	30 sec rest in between asana 10 × 30 sec = 5 min	20 min

Pranayama: Sectional Breathing (abdominal and thoracic), deep breathing	3 Pranayama × 2.5 min = 7.5 min	5 times/pranayama	50 sec rest in between Pranayama, 50 sec × 3 = 2.5 min	10 min

Total time: 45 minutes				

**Table 2:** Detailed yogic practice protocol for 2nd week (50 minute-schedule).

Name of yogic practices	Execution time	Repetitions	Recovery time	Total time
Suryanamaskar (3 days/week) OR	1.25 × 6 = 7.5 min	6 times	25 sec × 6 = 2.5 min	10 min
Shitilikarana Practices, Tadasana Practices (3 days/week)	2.84 × 3 = 8.25 min	3 times	35 sec × 3 = 1.75 min	

Kriya: Kapalbhati	45 strokes @ 1 stroke/sec = 3.75 min	5 times	15 sec rest in between each round = 1.25 min	5 min

Asana: Ardhahalasana, Ekpadapabanmuktasana, Bhujangasana, Ardhasalvasana, Yogmudra, Bakrasana, Janusirshasana, Lataral Chakrasana, Trikonasana, and Vadrasana	Holding time: 10 sec 10 Asana × 2 min = 20 min	1 times	30 sec rest in between Asana 10 × 30 sec = 5 min	25 min

Pranayama: hand in and out breathing, hand stretching breathing, and ankle stretching breathing	3 Pranayama × 2.5 min = 7.5 min	5 times/Pranayama	50 sec rest in between Pranayama, 50 sec × 3 = 2.5 min	10 min

Total time: 50 minutes				

**Table 3:** Detailed yogic practice protocol for 3rd week (60-minute schedule).

Name of yogic practices	Execution time	Repetitions	Recovery time	Total time
Suryanamaskar (3 days/week) OR	1.25 × 6 = 7.5 min	6 times	25 sec × 6 = 2.5 min	10 min
Shitilikarana Practices, Tadasana Practices (3 days/week)	2.84 × 3 = 8.25 min	3 times	35 sec × 3 = 1.75 min	

Kriya: Kapalbhati and	60 strokes @ 1 stroke/sec = 5 min 30 sec × 3 = 1.5 min	5 times	24 sec × 5 times = 2 min	7 + 3 = 10 min
Uddiyan		3 times	30 sec × 3 = 1.5 min	

Asana: Ardhahalasana, Ekpadapabanmuktasana, Bhujangasana, Ardhasalvasana, Yogmudra, Bakrasana, Janusirshasana, and Lataral	Holding time: 10 sec	2 times	12 × 15 sec × 2 = 6 min	25 min
Chakrasana, Trikonasana, Vadrasana, Matyasana, and Padmasana	12 Asana × 1.5 min = 18 min		1 min complete Savasana	

Pranayana: Suryabhedana, Chandrabhedana, and Anulome-vilome	3 Pranayana × 3 min = 9 min	5 times	20 sec rest in between Pranayana 20 × 3 = 1 min	10 min

Yogic relaxation: spinal practices	Five Variations 1 min × 5 = 5 min	One time each	—	5 min

Total time: 60 minutes				

**Table 4:** Detailed yogic practice protocol for 4th week (70-minute schedule).

Name of yogic practices	Execution time	Repetitions	Recovery time	Total time
Suryanamaskara	1 min × 8 = 8 min	8 times	15 sec × 8 times = 2 min	10 min

Kriya: kapalbhati Uddyan and Agnisara	80 strokes @ 1 stroke/sec = 6.40 min 30 sec × 3 × 2 = 3 min	5 times 3 times	40 sec × 5 times = 3.20 min 20 sec × 2 × 3 = 2 min	15 min

Asana: Ardhahalasana, Ekpadapabanmuktasana, Bhujangasana, Ardhasalvasana, Yogmudra, Bakrasana, Janusirshasana, Lataral Chakrasana, Trikonasana, Vadrasana, Matyasana, and Padmasana	Holding time: 10 sec 12 Asana × 1.5 min = 18 min	2 times	12 × 15 sec × 2 = 6 min 1 min complete Savasana	25 min

Pranayana: Anulome-Vilome, Shitali/Shifkari, and Bhastrika	3 Pranayana × 4 min = 12 min	5 times	1 min deep breathing in between Pranayama 1 × 3 = 3 min	15 min

Yogic relaxation	Relaxation through Savasana			5 min

Total time: 70 minutes				

**Table 5:** Detailed yogic practice protocol for 5th week (80-minute schedule).

Name of yogic practices	Execution time	Repetitions	Recovery time	Total time
Suryanamaskara	1 min × 12 = 12 min	12 times	15 sec × 12 = 3 min	15 min

Kriya: kapalbhati	60 strokes @ 2 stroke/sec = 2.5 min	5 times	30 sec × 5 times = 2.5 min	10 min
Uddyan and Agnisara (4 days/week) OR	30 sec × 3 × 2 = 3 min	3 times	20 sec × 2 × 3 = 2 min	
Jalaneti and Sutraneti 2 days/week	4 min × 2 = 8 min	1 times	1 min × 2 = 2 min	

Asana: Uttanpadasana, Pabanmuktasana, Naukasana, Matyasana, Bhujangasana, Salvasana, Naukasana (prone), Bakrasana, Usthrasana, Vajrasana, Yogmudra Pachimotthanasana, Tarasana, Trikomasana, and Katichakrasana	Holding time: 15 sec 15 × 1.5 min = 22.5 min	2 times	15 × 15 sec × 2 = 7.5 min	30 min

Pranayana: Anulome-vilome, Shitali/Shifkari, and Bhastrika	3 Pranayana × 4 min = 12 min	5 times	1 min deep breathing in between Pranayama 1 × 3 = 3 min	15 min

Meditation: A-U-M/chanting or complete Aum	Practice span: 10 min	Depend upon individual capacity	Complete relaxation during recitation	10 min

Total time: 80 minutes				

**Table 6:** Detailed yogic practice protocol for 6th and 7th week (90-minute schedule).

Name of yogic practices	Execution time	Repetitions	Recovery time	Total time
Suryanamaskar	40 sec × 12 = 8 min	12 times	10 sec × 12 = 2 min	10 min

Kriya: Kapalbhati	80 strokes @ 2 stroke/sec = 4 min	6 times	60 sec × 6 times = 6 min	15 min
Uddyan and Agnisara (3 days/week)	30 sec × 3 × 2 = 3 min	3 times	20 sec × 2 × 3 = 2 min	
Jalaneti and Sutraneti (2 days/week)	5 min × 2 = 10 min	1 times	2.5 min × 2 = 5 min	
Vaman Dauti (1 day/week)	10 min	1 times	5 min	

Asana: Uttanpadasana, Pabanmuktasana, Naukasana, Matyasana, Bhujangasana, Salvasana, Naukasana (prone), Bakrasana, Usthrasana, Vajrasana, and Yogmudra	Holding time: 20 sec	2 times	10 sec × 15 × 2 = 5 min	35 min
Pachimotthanasana, Tarasana, Trikomasana, and Katichakrasana	15 × 2 min = 30 min			

Pranayana: Anulome-violme, Shitali/Shitkari, Bhastrika, and Ujjai	4 Pranayama × 4 min = 16 min	5 times	1 min deep breathing in between Pranayama 1 min × 4 = 4 min	20 min

Meditation: A-U-M or complete “AUM” chanting	Practice span: 10 min	Depend upon individual capacity	Complete relaxation during recitation	10 min

Total time: 90 minutes				

**Table 7:** Detailed yogic practice protocol for 8th to 12th weeks (105-minute schedule, 1.45 hrs) combination-1 (2 days/week, Monday and Friday).

Name of yogic practices	Execution time	Repetitions	Recovery time	Total time
Kriya: Jalaneti, Sutraneti, and Vamandhauti	6 + 6 + 10 = 25 min	One	3 × 1 min = 3 min	25 + 5 = 30 min
Kapalbhati	60 strocks @ 2 strokc/sec = 2.5 min	5 Times	30 sec × 5 times = 2.5 min	

Suryanamaskar	25 sec × 15 = 6.25 min	15 Times	15 sec × 15 = 3.75 min	10 Min

Asana: Uttanpadasana, Pabanmuktasana, Sarbangasana, Halasana, Matyasana, Salvasana, Dhanurasana (modified), Bakrasana, Usthrasana, Suptavajrasana (modified), Yogmudra Pachimotthanasana, Tarasana, Trikomasana, and Katichakrasana	Holding time: 25 sec 15 × 2 min = 30 min	2 times	10 sec × 15 × 2 = 5 min	35 min

Pranayana: Anulome-violme, Shitali/Shitkari, Bhastrika, and Ujjai	4 Pranayama × 4 min = 16 min	5 times	1 min deep breathing in between Pranayama 1 min × 4 = 4 min	20 min

Meditation: A-U-M or complete “AUM” chanting	Practice span: 10 min	Depend upon individual capacity	Complete relaxation during recitation	10 min

Total time: 105 minutes				

**Table 8:** Detailed yogic practice protocol for 8th to 12th weeks (105-minute schedule, 1.45 hrs) combination-2 (2 days/week, Tuesday and Thursday).

Name of yogic practices	Execution time	Repetitions	Recovery time	Total time
Suryanamaskar	25 sec × 15 = 6.25 min	15 times	15 sec × 15 = 3.75 min	10 min

Kriya: Kapalbhati	60 strokes @ 2 stroke/sec = 2.5 min	5 times	30 sec × 5 times = 2.5 min	10 min
Uddyan and Agnisara	30 sec × 3 × 2 = 3 min	3 times	20 sec × 2 × 3 = 2 min	

Asana: Uttanpadasana, Pabanmuktasana, Sarbangasana, Halasana, Matyasana, Salvasana, Dhanurasana (modified), Bakrasana, Usthrasana, Suptavajrasana (modified), Yogmudra Pachimotthanasana, Tarasana, Trikomasana, and Katichakrasana	Holding time: 25 sec			35 min
	15 × 2 min = 30 min	2 times	10 sec × 15 × 2 = 5 min	

Pranayana: Anulome-violme, Shitali/Shitkari, Bhastrika, Ujjai, and Bharamari	5 Pranayama × 4 min = 20 min	5 times	1 min deep breathing in between Pranayama 1 min × 5 = 5 min	25 min

Meditation: Yog Nidra	Practice span: 25 min	—	Complete relaxation during the practice	25 min

Total time: 105 minutes				

**Table 9:** Detailed yogic practice protocol for 8th to 12th weeks (105-minute schedule, 1.45 hrs) combination-3 (2 days/week, Wednesday and Saturday).

Name of yogic practices	Execution time	Repetitions	Recovery time	Total time
Suryanamaskar	25 sec × 15 = 6.25 min	15 Times	15 sec × 15 = 3.75 min	15 Min

Kriya: Kapalbhati	80 strokes @ 2 stroke/sec = 4 min	6 times	60 sec × 6 times = 6 min	10 + 5 = 15 min
Uddyan and Agnisara	30 sec × 3 × 2 = 3 min	3 times	20 sec × 2 × 3 = 2 min	

Asana: Uttanpadasana, Pabanmuktasana, Viparitkaranisana, Halasana, Matyasana, Salvasana, Dhanurasana (modified), Bakrasana, Usthrasana, Suptavajrasana (modified), Yogmudra Pachimotthanasana, Tarasana, Trikomasana, and Katichakrasana	Holding time: 20 sec			45 min
	15 × 2.5 min = 37.5 min	3 times	10 sec × 15 × 3 = 7.5 min	

Pranayana: Anulome-violme, Shitali/Shitkari, Bhastrika, Ujjai, and Bharamari	5 Pranayama × 4 min = 20 min	5 times	1 min deep breathing in between Pranayama 1 min × 5 = 5 min	25 min

Meditation: A-U-M or complete “AUM” chanting	Practice span: 5 min	Depend upon individual capacity	Complete relaxation during recitation	5 min

Total time: 105 minutes				

**Table 10:** Baseline characteristics (independent “*t*” test).

Sl. number	Variables	Male (experimental pretest versus control pretest)	Female (experimental pretest versus control pretest)
1.	Body weight	*P* = 0.49	*P* = 0.99
2.	Body mass index	*P* = 0.40	*P* = 0.76
3.	hGH	*P* = 0.85	*P* = 0.44
4.	DHEAS	*P* = 0.71	*P* = 0.72

**Table 11:** General and endocrine variables of experimental and waitlist control group (Mean ± SD).

Components	Experimental group (yoga)			Control group (wait list)		
	Pretest	Midtest (pre. versus mid.)	Posttest (pre. versus post.)	Pretest	Midtest (pre. versus mid.)	Posttest (pre. versus post.)
Body weight (Kg) [male]	70.36 ± 14.14	69.93 ± 13.64	69.2 ± 13.64*	74.19 ± 15.81	74.2 ± 15.75	74.27 ± 15.70
Body weight (Kg) [female]	64.26 ± 8.87	63.27 ± 8.68**	62.4 ± 8.0***	64.21 ± 8.88	64.07 ± 8.87	63.05 ± 8.85
BMI (Kg/Mt^2^) [male]	24.33 ± 4.33	24.17 ± 4.22	23.93 ± 4.22*	25.64 ± 4.23	25.65 ± 4.02	25.68 ± 3.42
BMI (Kg/Mt^2^) [female]	25.89 ± 3.47	25.49 ± 3.66**	25.13 ± 3.48***	26.4 ± 3.13	26.34 ± 3.03	26.39 ± 2.75
hGH (ng/mL) [male]	0.46 ± 0.35	0.99 ± 0.51***	1.74 ± 0.09***	0.43 ± 0.39	0.43 ± 0.34	0.54 ± 0.03
hGH (ng/mL) [female]	0.39 ± 0.09	0.86 ± 0.12***	1.74 ± 0.28	0.34 ± 0.10	0.33 ± 0.08	0.36 ± 0.08
DHEAS (ng/mL) [male]	7.76 ± 3.48	8.97 ± 4.31*	12.17 ± 4.51***	8.17 ± 3.99	8.16 ± 3.41	7.98 ± 4.07
DHEAS (ng/mL) [Ffemale]	3.67 ± 1.46	4.85 ± 2.17**	5.45 ± 1.15***	3.33 ± 1.51	3.38 ± 1.52	3.42 ± 1.35

*P < 0.05, **P < 0.01, and ***P < 0.001; two tailed, *t*-test for paired data comparing the values at six weeks (midtest) versus baseline (pretest) and twelve weeks (posttest) versus baseline (pretest).

**Table 12:** Growth hormone (analysis of variance).

Source	Type III sum of squares	df	Mean square	*F *	Sig. (*P* value)	Eta squared
Male						
Within subjects factor (time: pretest, midtest, and posttest)	6.780	1	6.780	31.425	0.000	0.529
Between subjects factor (groups: experimental and wait list control)	8.354	1	8.354	16.678	0.000	0.373
Interaction time ∗ group	5.575	1	5.575	25.840	0.000	0.480
Error (within subjects factor)	6.042	28	0.216			
Error (between subjects factor)	14.025	28	0.501			

Female						
within subjects factor (time: pretest, midtest, and posttest)	4.371	1	4.371	47.499	0.000	0.856
Between subjects factor (groups: experimental and wait list control)	12.339	1	12.339	1.804	0.216	0.184
Interaction time ∗ group	3.570	1	3.570	38.795	0.000	0.829
Error (within subjects factor)	0.736	13	9.203			
Error (between subjects factor)	54.718	13	6.840			

**Table 13:** Dehydroepiandrosterone sulfate hormone (analysis of variance).

Source	Type III sum of squares	df	Mean square	*F *	Sig. (*P* value)	Eta squared
Male						
within subjects factor (time: pretest, midtest, and posttest)	69.768	1	69.768	21.748	0.000	0.437
Between subjects factor (groups: experimental and wait list control)	50.580	1	50.580	1.208	0.281	0.041
Interaction time ∗ group	82.415	1	82.415	25.690	0.000	0.478
Error (within subjects factor)	89.825	28	3.202			
Error (between subjects factor)	1171.933	28	41.855			

Female						
within subjects factor (time: pretest, midtest, and posttest)	0.792	1	0.792	23.303	0.001	0.744
Between subjects factor (groups: experimental and wait list control	1.587	1	1.587	70.123	0.000	0.898
Interaction time ∗ group	0.707	1	0.707	20.798	0.002	0.722
Error (within subjects factor)	0.272	13	3.399			
Error (between subjects factor)	0.181	13	2.263			
